# Early Postpartum Metabolic Heterogeneity Among Women Who Progressed to Type 2 Diabetes After Gestational Diabetes: A Prospective Cohort

**DOI:** 10.1002/dmrr.70027

**Published:** 2025-01-15

**Authors:** Saifur R. Khan, Julie A. D. Van, Zhang Xiangyu, Stacey E. Alexeeff, Babak Razani, Michael B. Wheeler, Erica P. Gunderson

**Affiliations:** ^1^ Department of Medicine Division of Cardiology University of Pittsburgh Pittsburgh Pennsylvania USA; ^2^ Vascular Medicine Institute University of Pittsburgh Pittsburgh Pennsylvania USA; ^3^ VA Medical Center Pittsburgh Pennsylvania USA; ^4^ Center for Immunometabolism University of Pittsburgh Pittsburgh Pennsylvania USA; ^5^ Departments of Physiology and Medicine University of Toronto Toronto Canada; ^6^ Division of Research Kaiser Permanente Northern California Pleasanton California USA; ^7^ Department of Health Systems Science Kaiser Permanente Bernard J. Tyson School of Medicine Pasadena California USA

**Keywords:** clustering, disease heterogeneity, gestational diabetes, incident type 2 diabetes, machine learning, metabolomics, prospective cohort

## Abstract

**Aims:**

Gestational diabetes mellitus (GDM) poses a significant risk for developing type 2 diabetes mellitus (T2D) and exhibits heterogeneity. However, understanding the link between different types of post‐GDM individuals without diabetes and their progression to T2D is crucial to advance personalised medicine approaches.

**Materials and Methods:**

We employed a discovery‐based unsupervised machine learning clustering method to generate clustering models for analysing metabolomics, clinical, and biochemical datasets. For this analysis, we selected 225 women who later developed T2D during the 12‐year follow‐up period from the cohort of 1010 women who returned to a non‐diabetic state at 6–9 weeks (study baseline) after a GDM pregnancy based on 2‐h 75 g research OGTTs. The optimal model was selected by assessing Bayesian Information Criterion values, class separation performance, and the potential for clinically distinguishable clusters, accounting for participant prenatal and early postpartum characteristics.

**Results:**

The selected model comprises three clusters: pancreatic beta cell dysfunction (cluster‐β: median HOMA‐B 161.3 and median HOMA‐IR 3.8), insulin‐resistance (cluster‐IR: median HOMA‐B 630.5 and median HOMA‐IR 16.8), and a mixed cluster (cluster‐mixed: median HOMA‐B 307.2 and median HOMA‐IR 8.6). These clusters are distinguishable based on postpartum blood test parameters such as glucose tolerance, HOMA indices, and fasting lipid profiles including triglycerides, leptin, HDL‐c, and adiponectin, as well as participant age and BMI. Metabolomic analysis identified unique molecular signatures for each cluster. However, the time to T2D onset was not statistically significant among the three clusters (*p* = 0.22).

**Conclusion:**

This study enhances our understanding of the heterogeneity of early postpartum metabolic profiles that characterise the future onset of T2D diabetes in a diverse cohort of women with GDM, revealing insights into distinct mechanisms and personalised intervention strategies for the prevention of T2D.

## 1 Introduction

Gestational diabetes mellitus (GDM) is a form of diabetes that manifests during pregnancy. Even though most women return to a non‐diabetic state after delivery, they have an increased and accelerated risk of developing overt type 2 diabetes (T2D). Studies have estimated that roughly 10% of pregnancies are affected by GDM and 30%–50% of these affected women will develop T2D within 10 years postpartum [1, 2]. Additionally, a history of GDM predisposes not only women to lifelong predisposition to cardiovascular and metabolic disorders but also their children [3, 4]. Therefore, GDM is a key determinant of diabetes across multiple generations.

Despite significant efforts, preventing diabetes progression after a GDM pregnancy has been challenging because of the highly heterogeneous risk profile. Maternal age, race and ethnicity, anthropometrics (including body mass index and excessive weight gain during pregnancy) along with various socioeconomic and lifestyle characteristics all interact and contribute to the pathophysiology of T2D after a GDM pregnancy [5, 6, 7]. Ultimately, these risk profiles underlie abnormalities in insulin action and secretion that likely existed before (often undetected and not diagnosed) and were further exacerbated after the index GDM pregnancy [5]. Elucidating these intricacies is crucial for the development and implementation of treatments using personalised medicine.

Subtyping risk populations may better dissect the heterogeneity of disease. Notably, Ahlqvist and colleagues identified five subtypes in the All New Diabetics In Scania (ANDIS) cohort for adult‐onset diabetes with distinct risks of complications, comorbidities, genetics, and response to treatment [8]. Given the highly homogeneous racial composition of the ANDIS cohort, further efforts have been undertaken to determine whether the subclassification can be replicated and used in other populations. To a lesser extent, GDM subtyping has been examined in recent years [9, 10]. While there is no consensus on definitions and outcomes, most studies have delineated women with GDM using the antepartum oral glucose tolerance testing as having sensitivity defects, secretion defects, or both. Whereas subtyping populations with either GDM or T2D is an ongoing endeavour, deciphering the specific and distinct profiles within women who progress to T2D after GDM remains to be established. We hypothesise that there are various clinical presentations and pathophysiological mechanisms associated with GDM‐to‐T2D progression. By establishing these profiles, we can better support clinical care, tailor research questions, and advance personalised medicine for the prevention of T2D after a GDM pregnancy.

In this analysis, we employed a model‐based clustering technique using two easily measured clinical variables, HOMA‐IR and HOMA‐B scores, at 6–9 weeks postpartum that identified three distinct clusters among 225 women with GDM from the Study of Women, Infant Feeding, and Type 2 Diabetes after Gestational Diabetes (SWIFT) who developed T2D during 12‐year prospective follow up [11]. Each GDM‐to‐T2D cluster was then extensively characterised for clinical, biochemical, and metabolomic traits, revealing distinct mechanisms of T2D progression.

## 2 Methods

### 2.1 SWIFT Study Prospective Research Cohort

The SWIFT study enroled a total of 1035 racially and ethnically diverse women with GDM (aged 20–45 years), diagnosed by 3‐h 100‐g OGTT per Coustan and Carpenter criteria, from 2008 to 2011. The participants comprised 35% Asian, 31% Hispanic, 8% Black, 24% White, and 2% mixed race/native. Briefly, eligibility criteria for enrolment of women into the early postpartum research cohort (study baseline 6–9 weeks postpartum) included 'no history of diabetes', 'no other serious health conditions', 'not receiving glucose tolerance altering medication', and 'no plan for another pregnancy', with the intention to exclusively or mostly breastfeed for at least 4 months or mostly formula feed from 1‐month post‐delivery. Participants provided written informed consent for three in‐person research visits at baseline and annually for up to two years post‐baseline, which each included a 2‐h 75‐g research oral glucose tolerance test (OGTT), biospecimen collection and assays, surveys, and other assessments per research protocol. Around 95% of the 1010 women who returned to a free of diabetes at baseline had repeated 2‐h research OGTTs and EHR clinical tests for diabetes during the 12‐year follow up period (2009–2020).

Trained research staff also assessed breastfeeding intentions during late pregnancy and again in the first month postpartum to determine cohort eligibility, utilising a validated questionnaire to measure the Infant Feeding Intention (IFI) score [12]. IFI scores were available for 698 of 1010 women in the SWIFT study. There was a graded increase in the mean IFI score with higher lactation intensity group at baseline (Supporting Information S1: Table 1). We therefore maintain that the BF groups reflected intention and intensity of lactation behaviour.

Plasma levels of insulin and glucose were performed by the University of Washington, Northwest Lipid Research Laboratories (Dr. Santica Marcovina), using samples collected from 2‐h 75‐g research OGTTs [11]. The study design and all procedures were approved by the KPNC Institutional Review Board in Oakland, CA, USA. Subsequent assays of stored specimens for metabolomics for this analysis were approved by the Office of Research Ethics at the University of Toronto.

All participants provided written informed consent for the SWIFT study (KPNC, Oakland, CA, USA), study data collection, use of the EHR data (ClinicalTrials.gov: NCT01967030), and future use of their research data and stored biospecimens in subsequent research studies. Details of the SWIFT study have been described elsewhere [1, 13].

### 2.2 Definition of Type 2 Diabetes

All participants consented to three in‐person research visits at 6–9 weeks (SWIFT baseline Visit 1), 1 year (Visit 2), and 2 years (Visit 3) postpartum. At these visits, women underwent 2‐h 75‐g OGTTs with repeat testing on a different day for elevated values for classification of incident T2D according to the American Diabetes Association criteria. Reclassification during the longer follow‐up period (> 32 months postbaseline) was performed using clinical data from the KPNC electronic health records system (laboratory tests, medical diagnoses from ICD codes, and medication use), reviewed by EPG every two years through October 2020 for the current analysis.

### 2.3 Study Design and Discovery‐Approach for Incident T2D Heterogeneity Identification

For this analysis, we selected all 226 women who developed new‐onset T2D after 6–9 weeks postpartum (baseline) during 12 years of follow‐up. One woman was excluded because of missing insulin measurements at baseline. Overall, these 225 women significantly differed in various clinical and biochemical characteristics at baseline from those who remained free of diabetes during the same follow‐up period (Supporting Information S1: Table 2). We therefore sought to describe the heterogeneity of the early postpartum profiles of these 225 women using clustering analysis. In doing so, we evaluated the common and distinct pathways responsible for the development of T2D within each cluster of women.

We employed a Gaussian finite mixture modelling algorithm named *mclust*, which is an unsupervised model‐based machine learning method [11, 14]. This approach included both with and without a feature selection strategy on different datasets within the same study group: metabolomics dataset, all clinical variables, biochemical variables, and their combination. The *mclust()* function constructs different models (i.e., EII, EVV, EEI, VII, VVI, VEE, EVE, VVE, VEI) from the data matrix by mixing components and the covariance parameterisation using the Bayesian Information Criterion (BIC), a penalised form of the log‐likelihood. The number of components (or clusters) is selected from the top models based on their BIC values before plateauing, ensuring no data overfitting. Details of *mclust* can be found elsewhere [11, 14]. The optimal cluster selection occurred in two phases. In the first phase, we compared the BIC of each clustering operation within the same kind of dataset. The clusters with the best BIC values (close to zero) for each kind of dataset were scrutinised for class separation/overlap both statistically and upon the integration of clinical and biochemical information.

### 2.4 Metabolomics

Metabolomics analyses were carried out using the AbsoluteIDQ p180 plate of the Biocrates system, which can analyse a total of 188 metabolites per sample. All assays were performed and assessed, without disclosure of group allocation, by the Analytical Facility for Bioactive Molecules (The Hospital for Sick Children, Toronto, ON, Canada). The significance of metabolomics data was corrected with multiple testing using FDR cut‐off < 0.05.

### 2.5 Statistical Analysis

Where relevant, we applied a nonparametric *t*‐test, and ANOVA to the clinical, socio‐demographic and biochemical research variables for the characterisation of each incident T2D case cluster. We identified clusters of T2D heterogeneity using a discovery‐based approach for the 225 cases. We employed a Gaussian finite mixture modelling algorithm named *mclust*, which is an unsupervised model‐based machine learning method.

## 3 Results

### 3.1 The Best Clustering Model Discovery for the Cohort

**FIGURE 1:**
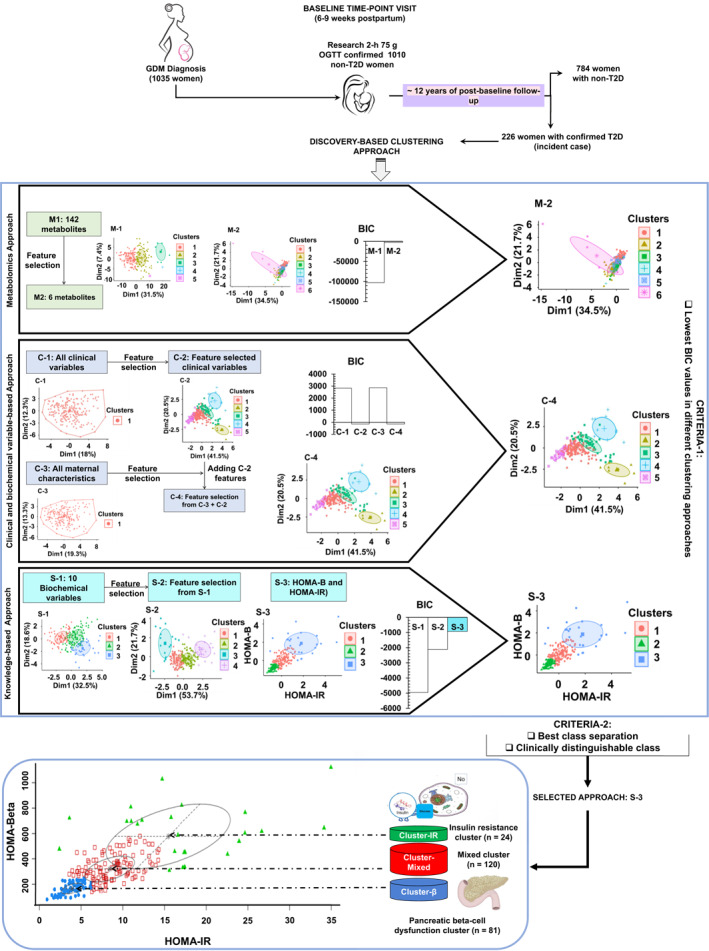
(A) Discovery‐based approach for clustering model selection. The flow diagram describes the study design, where metabolomics, clinical, and biochemical data from 225 incident cases (developing T2D within 12 years postpartum) out of the 1035 SWIFT participants underwent unsupervised model‐based clustering using the mclust algorithm with and without feature selection to identify heterogeneous groups. The optimal clustering model was selected based on two criteria: (I) the lowest BIC value in different clustering approaches, and (II) the best class separation both statically and clinically. The optimal model identified three clusters. These clusters are designated as Cluster‐β (representing participants with pancreatic beta‐cell dysfunction), cluster‐IR (representing participants with insulin resistance), and cluster‐mixed (exhibiting intermediate levels of both pancreatic beta‐cell dysfunction and insulin resistance).

In the first phase of model discovery, algorithms were run on metabolomics, clinical data, biochemical data, and all datasets together (Figure 1). However, the execution of all data together failed to provide any clusters, which are not shown here. We selected three models by comparing the BIC values within the same dataset, either metabolomics/combination of clinical data or biochemical data/knowledge‐based biochemical data. In the next phase, we thoroughly examined the data for overlapping and distinguishable biochemical variables. Ultimately, we selected the S3 model, which comprises HOMA‐B and HOMA IR, as the most suitable model (Figure 1).

### 3.2 Identification of Incident Case Clusters

The S3 model identified three distinct incident T2D clusters within a cohort of 225 women who developed incident T2D within 12 years post‐delivery (Figure 1). The resulting clusters are as follows: Cluster‐β comprises 81 incident T2D cases, Cluster‐mixed includes 120 incident T2D cases, and Cluster‐IR consists of 24 incident T2D cases.

**FIGURE 2:**
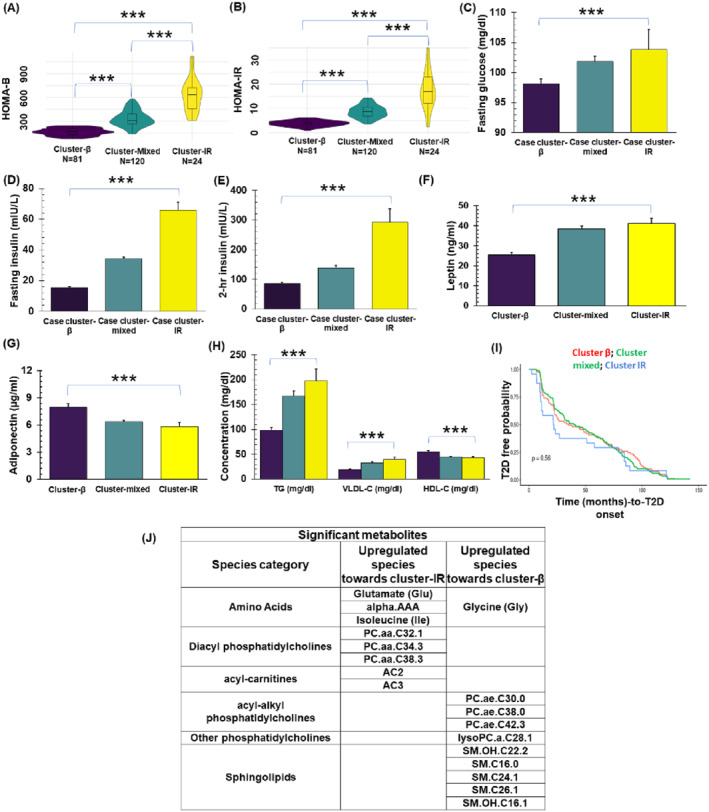
Heterogeneous group identification and characterisation using mean ± SEM. (A–B) The violin box plots of HOMA‐B and HOMA‐IR values between these three clusters respectively (*p < 0.05, **p < 0.01, and ***p < 0.001). (C–H) A comparison of some major clinical variables (i.e., fasting glucose, 2h insulin, fasting insulin, triglyceride, VLDL cholesterol, HDL cholesterol, leptin, and adiponectin) between these clusters (*p < 0.05, **p < 0.01, and ***p < 0.001). (I) Time to T2D onset analysis between clusters. (J) ANOVA identified 18 significant metabolites at the baseline between these clusters. These metabolites were presented here in a tabular form. The significance of metabolomics data was corrected with multiple testing using FDR cut‐off <0.05.

Cluster‐β demonstrates comparatively the poorest pancreatic beta‐cell function (i.e., HOMA‐B, Median [IQR]: 161.3 [126.8‐202.4]), yet the highest insulin sensitivity (i.e., HOMA‐IR, Median [IQR]: 3.82 [2.97‐4.59]) (Figure 2A–B). As a result, we refer to this cluster as the 'pancreatic beta‐cell dysfunction' group. On the other hand, Cluster‐IR exhibited comparatively the best pancreatic beta‐cell function (i.e., HOMA‐B, Median [IQR]: 630.5 [456.1‐722.3]) but the lowest insulin sensitivity (i.e., HOMA‐IR, Median [IQR]: 16.8 [12.1‐22.9]) (Figure 2A–B). Thus, we label this cluster as the 'insulin resistance' group. Cluster‐mixed exhibits a moderate level of both pancreatic beta‐cell function (i.e., HOMA‐B, Median [IQR]: 307.2 [263.5‐389.3]) and insulin sensitivity (i.e., HOMA‐IR, Median [IQR]: 8.6 [6.7‐10.5]) (Figure 2A–B). Consequently, Cluster‐mixed is categorised as a ‘Mixed group’.

### 3.3 Socio‐Demographic and Phenotypic Characteristics of Case Clusters

We examined a racially and ethnically diverse cohort of 225 women who later progressed to T2D (31.0% Asian, 12.0% Black, 37.6% Hispanic, 0.9% mixed race/native, and 18.5% White, Supporting Information S1: Table 2). Table 1 outlines the pre‐pregnancy and baseline characteristics of the three clusters. The racial and ethnic distributions of these women across different clusters were not significantly different. The mean age (mean ± SD, years) of participants at study baseline in cluster‐β (35.5 ± 4.3) is significantly older than those in both cluster‐mixed (33.5 ± 5.1) and cluster‐IR (30.9 ± 6.0) groups (*p* < 0.001). Conversely, there are higher levels of overall and central adiposity among the three clusters, as evidenced by differences in pre‐pregnancy BMI (*p* < 0.001), and both BMI and waist circumference at the study baseline (6–9 weeks postpartum) (*p* < 0.001). Cluster‐β′s mean (mean ± SD) pre‐pregnancy BMI (30.5 ± 7.1) is significantly lower than BMI for cluster‐mixed (35.4 ± 8.4) and cluster‐IR (36.1 ± 6.8) (*p* < 0.001). Similarly, cluster‐β exhibits significantly lower early postpartum BMI, and waist circumference compared to cluster‐mixed and cluster‐IR (all *p* < 0.001). Notably, GDM severity was similar across clusters, with no statistically significant differences in parity (*p* = 0.422), treatment for GDM (*p* = 0.181), gestational age at GDM diagnosis (*p* = 0.064), glycaemic control during pregnancy (*p* = 0.17), and family history of diabetes (*p* = 0.169). We also showed that the efficacy of treatment by medication and/or diet alone on glycaemic control was not significantly different across clusters (*p* = 0.053, Supporting Information S1 Table 3).

**TABLE 1:** Participant characteristics who developed incident diabetes after gestational diabetes by cluster.

Characteristics	Cluster‐β (*n* = 81)	Cluster‐mixed (*n* = 120)	Cluster‐IR (*n* = 24)	*p*‐value
Socio‐demographic				
Age (years) at delivery	35.5 (4.3)	33.5 (5.1)	30.9 (6.0)	< 0.001
Race and ethnicity, *n* (%)				0.371
Asian	30 (37.0)	35 (29.2)	4 (16.7)	
Black	9 (11.1)	15 (12.5)	3 (12.5)	
Hispanic	24 (29.6)	48 (40.0)	13 (54.2)	
Mixed race/Native	0 (0)	2 (1.7)	0 (0)	
White	18 (22.2)	20 (16.7)	4 (16.7)	
Pre‐pregnancy BMI (kg/m^2^)	30.5 (7.1)	35.4 (8.4)	36.1 (6.8)	< 0.001
Family history of diabetes, *n* (%)	51 (63.0)	72 (60.0)	10 (41.7)	0.169
Pregnancy				
Parity at index birth, *n* (%)				0.422
Primiparous (1 birth)	30 (37.0)	34 (28.3)	7 (29.2)	
Biparous (2 births)	23 (28.4)	46 (38.3)	11 (45.8)	
Multiparous (> 2 births)	28 (34.6)	40 (33.3)	6 (25.0)	
Gestational age at GDM diagnosis (weeks)	21.4 (8.4)	22.1 (8.3)	25.9 (8.6)	0.064
Pregnancy oral glucose tolerance test (3‐h 100 g OGTT)				
Fasting glucose level (mg/dL)	96.7 (14.0)	99.2 (11.6)	100.9 (15.1)	0.261
1‐h post glucose level (mg/dL)	208.8 (25.8)	207.0 (25.0)	205.3 (31.0)	0.810
2‐h post glucose level (mg/dL)	182.8 (32.6)	177.5 (32.5)	192.5 (31.0)	0.107
3‐h post glucose level (mg/dL)	130.1 (38.9)	127.5 (32.5)	143.9 (32.2)	0.114
3‐h OGTT z‐score sum of glucose values	1.1 (3.2)	1.0 (2.9)	2.1 (3.7)	0.284
GDM prenatal treatment, *n* (%)				0.181
Diet only	39 (48.2)	52 (43.3)	9 (37.5)	
Oral medications	35 (43.2)	57 (47.5)	9 (37.5)	
Insulin	7 (8.6)	11 (9.2)	6 (25.0)	
Glycemic control during pregnancy^a^				0.17
Optimal	47 (64.4)	54 (51.4)	7 (46.7)	
Suboptimal	26 (35.6)	51 (48.6)	8 (53.3)	
Research visit at 6‐9 Weeks postpartum (study baseline 2008‐2011)				
Glucose tolerance from 2‐h 75 g OGTT, *n* (%)				0.025
Normal	35 (43.2)	36 (30.0)	9 (37.5)	
IFG only	20 (24.7)	47 (39.2)	7 (29.2)	
IGT only	17 (21.0)	14 (11.7)	1 (4.2)	
IFG + IGT	9 (11.1)	23 (19.2)	7 (29.2)	
Lactation intensity categories, *n* (%)				0.006
Exclusive lactation	19 (23.5)	15 (12.5)	1 (4.2)	
Mostly lactation	40 (49.4)	46 (38.3)	7 (29.2)	
Mostly formula/Mixed/Inconsistent	12 (14.8)	35 (29.2)	8 (33.3)	
Exclusively formula	10 (12.4)	24 (20.0)	8 (33.3)	
Physical activity, met‐h/week, median (IQR)	44.5 (31.6, 66.9)	50.8 (36.9, 66.5)	44.7 (33.1, 56.1)	0.532
Diet, intake from fat, % kcal	26.0 (8.7)	26.5 (6.7)	28.3 (9.1)	0.457
Diet, intake from fibre, kcal	1.03 (0.34)	1.02 (0.37)	1.00 (0.39)	0.943
Anthropometry				
Weight (kg)	76.8 (19.3)	91.4 (19.3)	93.4 (18.0)	<0.001
Height (cm)	159.7 (6.7)	159.8 (8.0)	160.8 (6.1)	0.817
BMI (kg/m^2^)	29.9 (6.3)	35.6 (7.6)	36.0 (5.5)	<0.001
BMI Categories, *n* (%):				<0.001
Underweight or Normal (< 25 kg/m^2^)	19 (23.5)	4 (3.3)	1 (4.2)	
Overweight (25–29.9 kg/m^2^)	25 (30.9)	26 (21.7)	1 (4.2)	
Obese (≥ 30 kg/m^2^)	37 (45.7)	90 (75)	22 (91.7)	
Waist circumference (cm)	90.5 (12.8)	101.0 (14.3)	104.5 (11.8)	<0.001
Plasma, 2‐h 75 g research OGTT at 6‐9 weeks postpartum				
Fasting glucose (mg/dL)	98.2 (7.5)	101.6 (9.6)	103.2 (16.1)	0.022
2‐h post‐load glucose (mg/dL)	132.8 (34.8)	126.0 (29.5)	131.0 (32.1)	0.414
Fasting insulin (μU/mL), median (IQR)	16.3 (12.5, 18.2)	33.7 (26.7, 40.6)	62.5 (52.0, 75.9)	<0.001
2‐h post‐load insulin (μU/mL), median (IQR)	76.9 (58.1, 107.8)	117.6 (84.9, 158.3)	212.3 (164.0, 344.0)	<0.001
HOMA‐IR, median (IQR)	3.8 (3.0, 4.6)	8.6 (6.7, 10.5)	16.8 (12.1, 22.9)	<0.001
HOMA‐B, median (IQR)	161.3 (126.8, 202.4)	307.2 (263.5, 389.3)	630.5 (456.1, 722.3)	<0.001
Fasting triglycerides (mg/dL), median (IQR)	88 (64, 116)	131 (94, 200)	158 (112, 271)	<0.001
Fasting HDL‐C (mg/dL)	55.9 (12.8)	44.7 (10.5)	42.9 (12.6)	<0.001
Fasting LDL‐C (mg/dL)	126 (30)	121 (30)	116 (27)	0.325
Fasting adiponectin (ug/mL), median (IQR)	7.4 (5.5, 8.8)	6.0 (5.0, 7.4)	5.3 (4.7, 6.9)	<0.001
Fasting leptin (ng/mL)	23.5 (13.3)	38.7 (14.3)	41.2 (13.1)	<0.001
During post‐baseline to end of follow up through 2020				
Follow up time (months), median (IQR)	43.5 (13.1, 89.7)	35.8 (18.7, 79.7)	21.8 (10.3, 80.4)	0.22

*Note:* (*p*‐values: Chi‐squared used for categorical variables, ANOVA used for continuous, and Kruskal‐Wallis used for medians). Mean (SD) or *n* (%) unless otherwise noted as Median (IQR) for skewedness of variables.

Abbreviations: IFG, impaired fasting glucose; IGT, impaired glucose tolerance; 2‐h post‐load glucose.

^a^ There were missing values for glycaemia control during pregnancy (*n* = 32).

### 3.4 Fasting Biochemical Variables for the Case Clusters

The three incident T2D clusters showed significant differences across metabolic research parameters, including fasting plasma glucose and insulin, 2‐h post‐load plasma insulin, fasting plasma triglycerides, HDL‐cholesterol, VLDL‐cholesterol, adiponectin, and leptin (Figure 2C–H, Table‐1). In essence, the requirement for insulin increased with the degree of insulin resistance following the sequence of cluster‐β < cluster‐mixed < cluster‐IR. Despite cluster‐IR requiring the highest level of insulin, this cluster also exhibited a notable worsening of fasting glucose (Table‐1). Furthermore, baseline levels of fasting triglycerides and VLDL‐cholesterol were elevated in the incident T2D cases with more severe insulin resistance. For instance, cluster‐IR had significantly higher levels of triglycerides and VLDL‐cholesterol compared to both cluster‐mixed and cluster‐β (Table‐1). The opposite trend, supported by statistical significance (*p* < 0.001), was observed for fasting HDL‐cholesterol and adiponectin concentrations (Table‐1). Regarding the adipokine Leptin, both cluster‐IR and cluster‐mixed exhibited significantly higher levels compared to cluster‐β (Table‐1).

### 3.5 Breastfeeding Intensity Variations and Time to T2D Onset Comparison

Given that lactation is recognized for its protective roles in T2D progression, we assessed lactation intensity at baseline for each cluster phenotype. Notably, 72.9% of incident T2D cases in cluster‐β were found exclusively or mostly in the lactation intensity group, compared to 50.8% in cluster‐mixed and 33.4% in cluster‐IR (Table‐1) (*p* = 0.006). This underscores significantly higher lactation intensity in cluster‐β when compared to the other clusters.

To evaluate differences in the time from 6 to 9 weeks postpartum OGTT after GDM to incident T2D with pancreatic *β*‐cell dysfunction or insulin resistance, we compared the median time to T2D onset among the clusters. The median times‐to‐onset were similar for cluster‐β and cluster‐mixed, approximately 43 and 35 months, respectively. In contrast, a higher proportion of cluster‐IR developed T2D earlier, with a median of about 22 months (Table‐1). However, these overall differences in the time to T2D onset were not statistically significant (*p* = 0.22) (Figure 2I).

### 3.6 Assessing Cluster Classification Over Time

We used the original clustering model to predict new cluster classification in women who underwent at least 1 research 2‐h 75‐g oral glucose tolerance test during one of the two annual follow‐up visits. Since 57 did not have any follow‐up measurements, 168 out of the 225 women in the cohort were examined and reclassified. Follow‐up HOMA‐IR and HOMA‐B scores at 1–2 years postpartum were used as inputs for the original clustering model. For this analysis, we observed that around half of the women maintained the same cluster status (Supporting Information S1: Figure 1). The most dominant direction of cluster movement was from cluster‐β to cluster‐mixed to cluster‐IR, accounting for 75% of women who were reclassified to a different cluster after 1–2 years postpartum.

### 3.7 The Molecular Characteristic of the Early‐Stage T2D Pathophysiology Between Case Clusters

**FIGURE 3:**
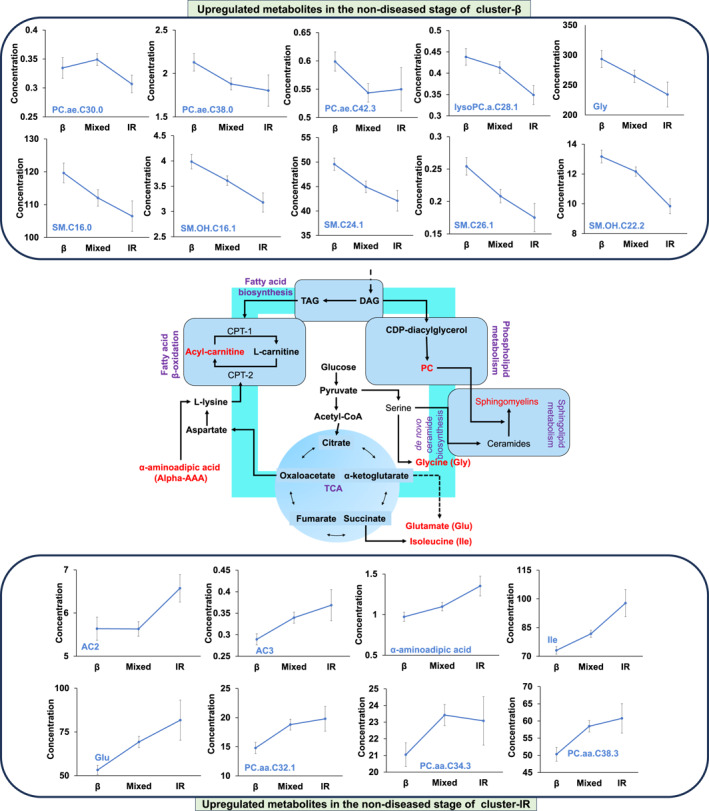
Metabolism roadmap for the three clusters. Clusters were identified with distinct metabolite alterations, primarily categorised within key pathways: sphingolipid metabolism, phospholipid metabolism, fatty acid metabolism (particularly fatty acid beta‐oxidation), and amino acid metabolism. Cluster‐β (pancreatic beta‐cell dysfunction): elevated sphingolipids, increased acyl‐alkyl phosphatidylcholines, raised lysophosphatidylcholine, and higher glycine levels. Cluster‐IR (insulin resistance): enhanced diacyl phosphatidylcholines, elevated acyl‐carnitines, increased isoleucine levels, and raised glutamate concentrations. Cluster‐mixed (intermediate characteristics): metabolites present at intermediate concentrations. These alterations in metabolites offer insights into the distinct metabolic profiles of the non‐disease stage of the clusters.

The baseline fasting plasma metabolomics data were harnessed to discern distinct early‐stage pathophysiological profiles among the case clusters at the molecular level. Notably, 18 metabolites displayed significant differences between the incident T2D case clusters, encompassing phospholipids, sphingolipids, carnitines, and amino acids (Figure 2J). Specifically, seven phospholipids exhibited significant alterations, categorised as three diacyl phosphatidylcholines (PC aa C32:1, PC aa C34:3, PC aa C38:3), three acyl‐alkyl phosphatidylcholines (PC ae C30:0, PC ae C38:0, PC ae C42:3), and one lysophosphatidylcholine (lysoPC a 28:1). Interestingly, the concentration trend of altered diacyl phosphatidylcholines increased from case cluster‐β to case cluster‐IR, while the reverse was observed for altered acyl‐alkyl phosphatidylcholines and lysophosphatidylcholine (Figure 2, 3, Supporting Information S1: Figure 2). Additionally, five sphingolipids displayed significant differences between case clusters, including SM C16:0, SM(OH)C16:1, SM(OH)C22:2, SMC24:1, and SMC26:1. Notably, the plasma concentration trend of altered sphingolipids declined from case cluster‐β to cluster‐IR (Figure 2, 3, Supporting Information S1: Figure 3). Moreover, significant differences were observed among the three case clusters in terms of blood concentrations of two acetylcarnitines (AC2 and AC3) and four amino acids (glutamate, glycine, *α*‐aminoadipic acid, and isoleucine) (Figure 2, 3, Supporting Information S1: Figure 4). These alterations displayed an ascending concentration trend for acetylcarnitine and most amino acids (except glycine) from case cluster‐β to cluster‐IR. An exception was observed for glycine, which displayed an opposite trend, being highest in cluster‐β.

## 4 Conclusions

In this study, we identified three clusters of women who developed T2D within 12 years postpartum of a GDM pregnancy using a discovery‐based, model‐driven, machine learning clustering algorithm. HOMA‐IR and HOMA‐B scores at 6–9 weeks postpartum served as the optimal input variables for the final clustering model. The three clusters were defined by pancreatic beta‐cell dysfunction (cluster‐β), insulin resistance (cluster‐IR), and mixed presentations (cluster‐mixed).

Notably, over 50% of the 225 women in the current analysis were classified into cluster‐mixed with intermediate characteristics of cluster‐β (*n* = 81) and cluster‐IR (*n* = 24). This observation aligns with previous clustering studies where a hard clustering rule was applied to women with GDM, leading to the identification of three similar clusters during pregnancy [15, 16, 17]. Overall, this suggests that early postpartum GDM profiles and longer‐term progression to T2D after GDM exhibit a similar kind of disease heterogeneity. As expected, the clusters identified in this study are clinically distinguishable based on several baseline glucometric research parameters, such as fasting and 2‐h post‐load levels of glucose and insulin. We also observed that women in cluster‐β were typically older and leaner than those in the cluster‐IR group. In contrast, women from cluster‐IR presented with a more pathophysiologic lipid biochemistry profile, having the highest fasting triglyceride levels and lowest HDL‐cholesterol levels among all clusters. Interestingly, these clusters did not reveal any significant racial or ethnic patterns, suggesting that the underlying mechanisms may be common across multiple demographic groups.

From a behavioural perspective, we observed higher intensity of lactation for a higher percentage of women in cluster‐β. Where the median time to T2D progression for cluster‐β women was longer (43 weeks), in contrast to women in cluster‐IR with shorter median time (22 weeks). Previously, strong protective associations for higher lactation intensity and longer duration were found in SWIFT; a 50% reduction in the relative risk of two‐year incidence of incident T2D [1]. Other beneficial effects of exclusive or full lactation include more favourable metabolic profiles at baseline, including lower fasting insulin and glucose levels while maintaining higher HDL‐C levels [1, 13, 18]. However, differences in lactation intensity among the clusters at study baseline are consistent with our previous longitudinal cohort studies, but this analysis did not include women who did not progress to T2D during the same 12‐year follow up period. Additionally, numerous factors other than physiological aspects may impede successful lactation, specifically higher rates of obstetrical complications and lower breastfeeding support among women with obesity, as well as barriers social and cultural factors.

Metabolomic profiling was integrated to offer a more comprehensive characterisation of the metabolic differences underlying beta cell dysfunction and insulin resistance during the non‐diseased postpartum period in incident T2D. In total, we identified 18 metabolites that exhibited significant differential alterations among the three clusters, primarily categorised within key pathways: sphingolipid metabolism, phospholipid metabolism, fatty acid metabolism (particularly fatty acid beta‐oxidation), and amino acid metabolism. We recently demonstrated that diminished sphingolipid metabolism may lead to pancreatic beta‐cell dysfunction [19]. Griess and colleagues further identified that pancreatic *β*‐cell‐specific CERS2 function is essential for pancreatic beta‐cell function [20]. Interestingly, we observed a significant increase in several sphingolipids, including CERS2‐related sphingomyelins (i.e., SM[OH]C22:2, SMC24:1, and SMC26:1), in the non‐diseased stage of cluster‐β, which may suggest a defensive role against pancreatic beta‐cell dysfunction. This analysis suggests that impairments in sphingolipid metabolism may already be at play during this early postpartum period. Importantly, a key metabolite, glycine, was significantly higher in cluster‐β and may play a vital role in ameliorating oxidative stress‐induced pancreatic beta‐cell dysfunction by enhancing plasma antioxidants (glutathione, catalase, and superoxide dismutase) [21]. Additionally, it improves pancreatic beta‐cell mitochondrial degeneration and insulin granule degranulation processes, both of which decline with ageing [22]. Furthermore, glycine is recognized for its capacity to enhance insulin secretion from pancreatic beta‐cells through the glycine‐insulin autocrine feedback loop mechanism [23, 24]. Altogether, the higher concentration of glycine at the non‐disease stage of incident beta‐cell dysfunction (cluster‐β) highlights its protective role.

Insulin resistance appears to be associated with higher levels of acylcarnitines. These metabolites facilitate the transportation of long‐chain fatty acids into the mitochondria for fatty acid beta‐oxidation (i.e., a part of the fatty acid metabolism pathway). Given that cluster‐IR features a more obese and hyperlipidemic profile, the increase in acylcarnitine levels may reflect as a protective mechanism against insulin resistance [25]. This cluster also had higher levels of three amino acids. Among them, isoleucine is known for its protective role against insulin resistance by stimulating insulin‐independent glucose uptake in skeletal muscle cells [26]. Glutamate, however, has been adversely associated with the development of T2D [27]. Thus, the significant increase in these amino acids observed in cluster‐IR suggests their protective and contributory roles in the early disease stage of insulin resistance. On the other hand, *α*‐aminoadipic acid, an oxidation product of lysine, serves as a predictive biomarker for insulin resistance in obesity and may appear in circulation due to age‐related tissue degradation [28, 29]. Hence, *α*‐aminoadipic acid could function as an early ageing marker of insulin resistance for T2D progression and may not possess any protective roles.

Our analysis has strengths and limitations. We examine a unique progression towards diabetes that enables a focused examination of women. We leveraged a racially and ethnically diverse subset of women from the SWIFT cohort, who were followed up from GDM pregnancy for up to 12 years postpartum. Importantly, none of the incident T2D cases were clinically identified as MODY (Maturity‐onset diabetes of the young) or type 1 diabetes group based on the medical record view for KPNC diagnoses of diabetes, and all 225 women in the current analysis underwent research oral glucose tolerance testing at 6–9 weeks postpartum for accurate and reliable classification as well as HOMA assessments. To date, this study is the first to specifically examine and describe the subset of women who progressed from GDM to T2D. Future studies encompassing the broader cohort of women with GDM are indeed required to adequately address and dissect the risk profiles. Another limitation of the study is the small numbers, which likely led to the lack of significant differences observed among the clusters in time to T2D. Alternatively, this may suggest that both beta cell dysfunction and insulin resistance play distinct roles in driving T2D progression after GDM pregnancy. Consequently, interventions should prioritise addressing both aspects of pathophysiology.

In conclusion, we confirm early postpartum heterogeneity in metabolic profiles in women with GDM who later develop new onset T2D. These clusters were defined on clinically accessible variables, although insulin is not routinely measured in clinical settings. Finally, the clusters represented unique lipid profiles ascertained from biochemical and metabolomic analyses, and clinical phenotypes related to age and obesity. Our study offers key insights into the heterogeneous profiles at 6–9 weeks postpartum among a diverse sample of women who progress to T2D within 12 years after a GDM pregnancy. These profiles may help tailor the discovery and implementation of personalised medicine for T2D prevention in women.

## Author Contributions

SRK, MBW and EPG conceptualised and designed the research work executed by SRK. SRK and JADV wrote the manuscript, edited by all. All authors reviewed the manuscript and gave final approval of the version to be published.

## Ethics Statement

Requests to access the dataset from qualified researchers trained in human subjects' confidentiality protocols may be sent to Dr. Erica P. Gunderson, Principal Investigator, at the Division of Research, email: erica.gunderson@kp.org. The patient data are owned by the Kaiser Foundation Health Plan, Inc., Kaiser Foundation Hospitals, Inc., and The Permanente Medical Group, Inc. Because of their third‐party rights, it is not possible to make the data publicly available without restriction.

## Consent

The study participants provided written informed consent for all study research procedures, including use of biospecimens, research visit data, and collection of participant data from the KP electronic health records (EHR) system, and the data analysis for future studies. The Kaiser Permanente Northern California (KPNC) Institutional Review Board approved the Study of Women, Infant Feeding and Type 2 Diabetes after Gestational Diabetes (SWIFT) design, research protocol and procedures, and this data analysis. This analysis followed the Strengthening the Reporting of Observational Studies in Epidemiology (STROBE) guidelines.

## Conflicts of Interest

The authors declare no conflicts of interest.

### Peer Review

The peer review history for this article is available at https://www.webofscience.com/api/gateway/wos/peer-review/10.1002/dmrr.70027.

## Supporting information

Supporting Information S1

## Data Availability

The data that support the findings of this study are available on request from the corresponding author. The data are not publicly available due to privacy or ethical restrictions.
