# Supplementary material for: Early Postpartum Metabolic Heterogeneity Among Women Who Progressed to Type 2 Diabetes After Gestational Diabetes: A Prospective Cohort
Source: Diabetes Metab Res Rev. 2025 Jan 15;41(1):e70027. doi: 10.1002/dmrr.70027 (PMC11733828; doi:10.1002/dmrr.70027)
Supplement: Supplementary file 1 — Supporting Information S1 [file DMRR-41-e70027-s001.docx]

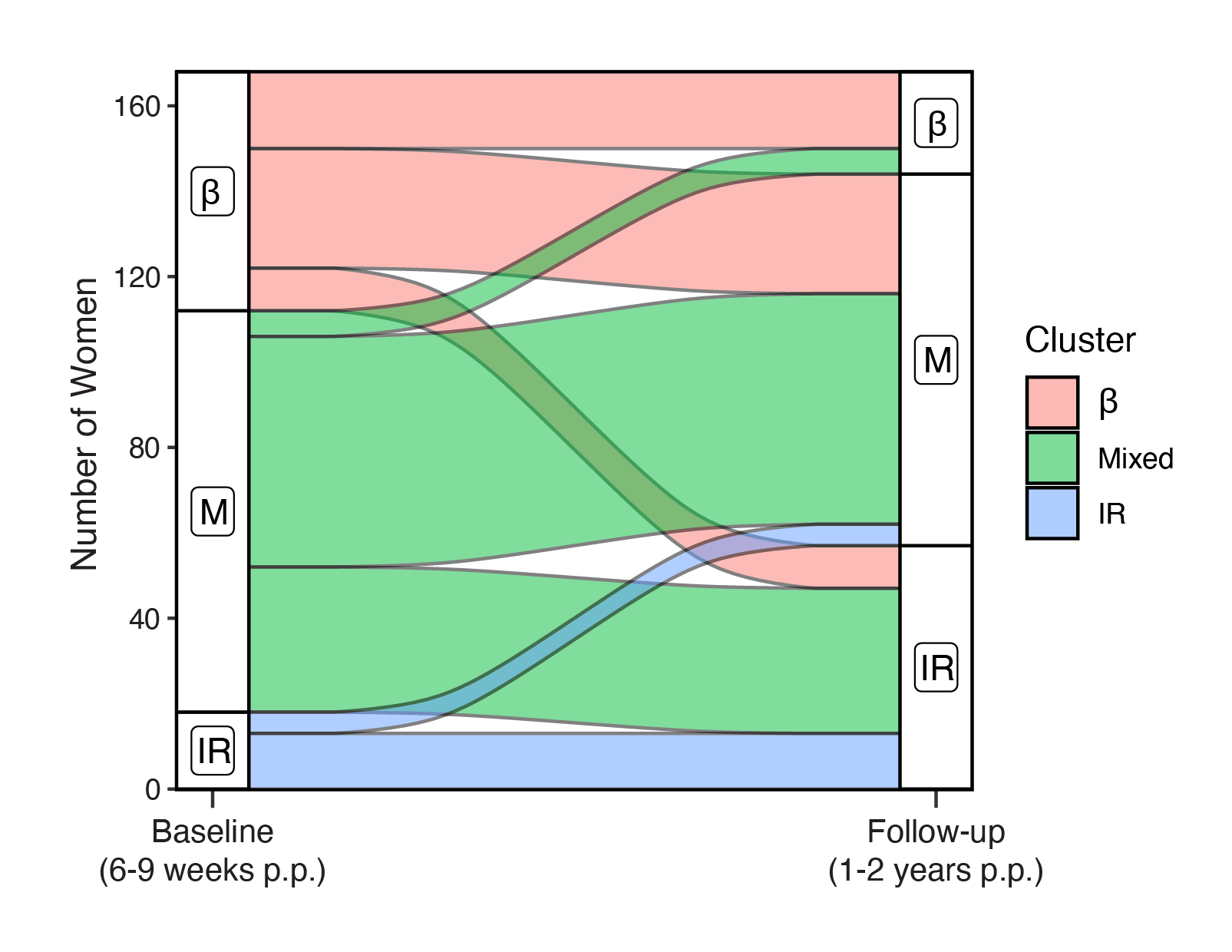


Figure S1. Alluvial plot showing movement between cluster identities of 168 women with follow-up testing from baseline to 1-2 years postpartum.


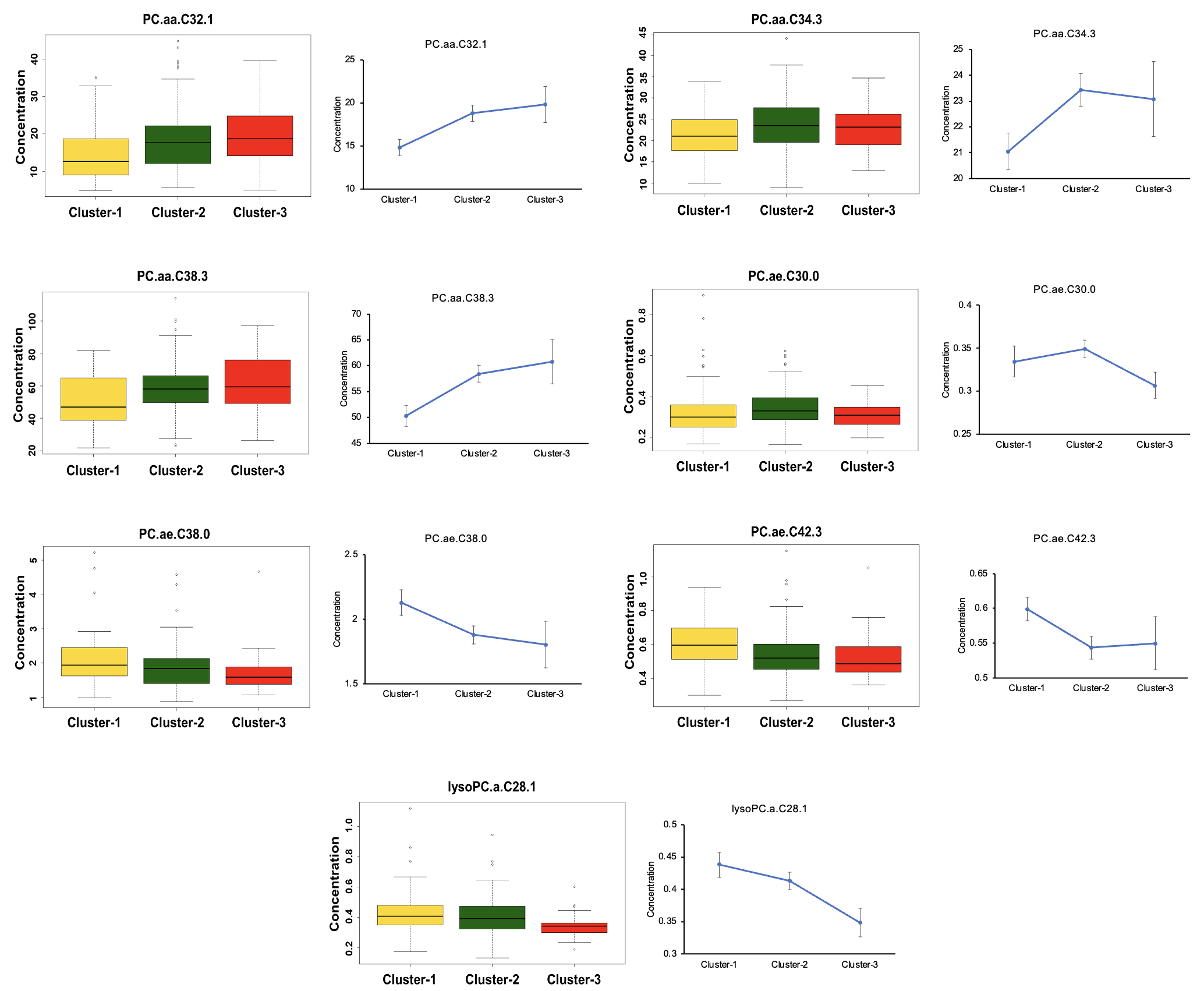


Figure S2. Concentrations of select phospholipids.


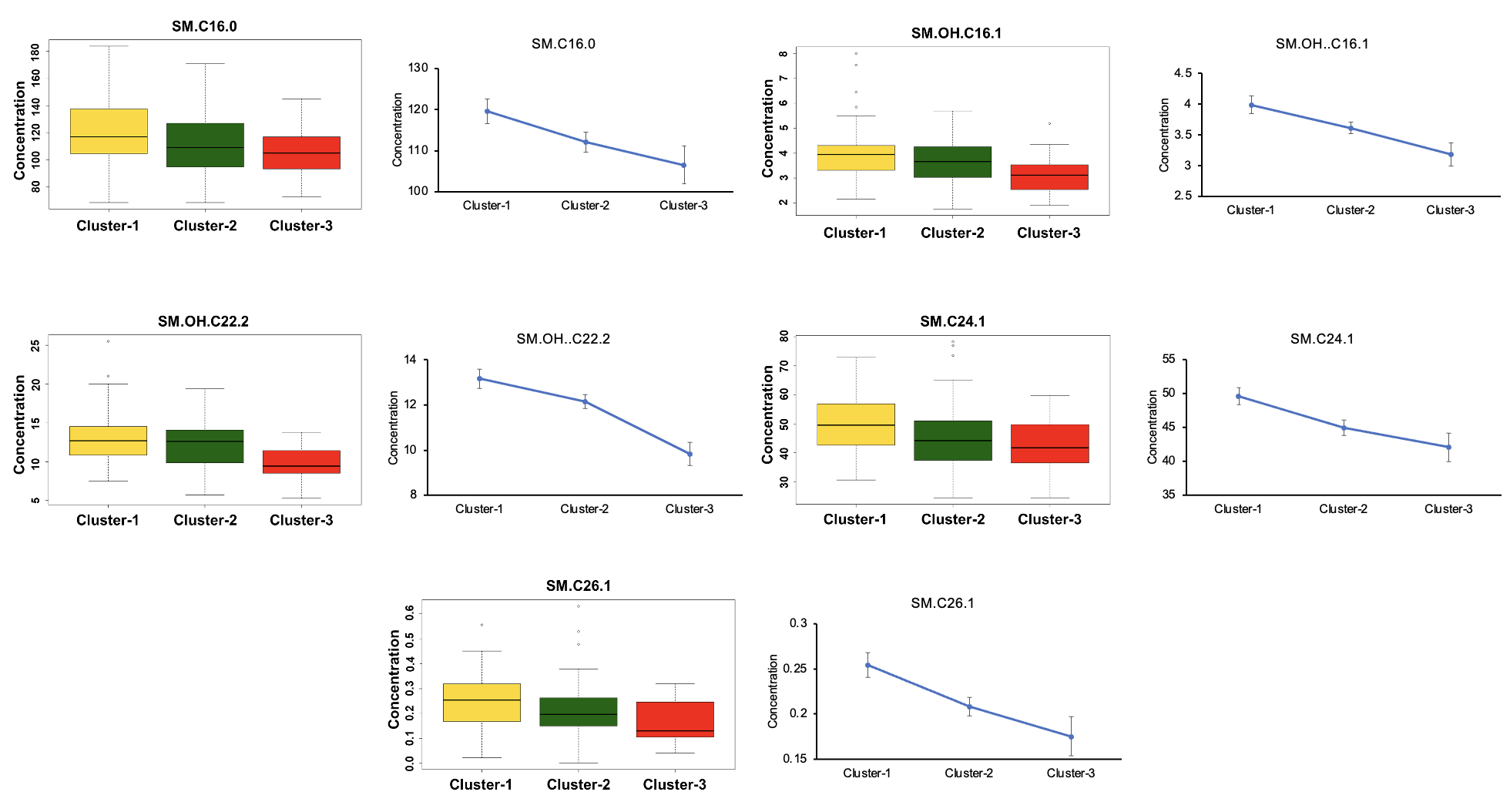


Figure S3. Concentrations of select sphingolipids.


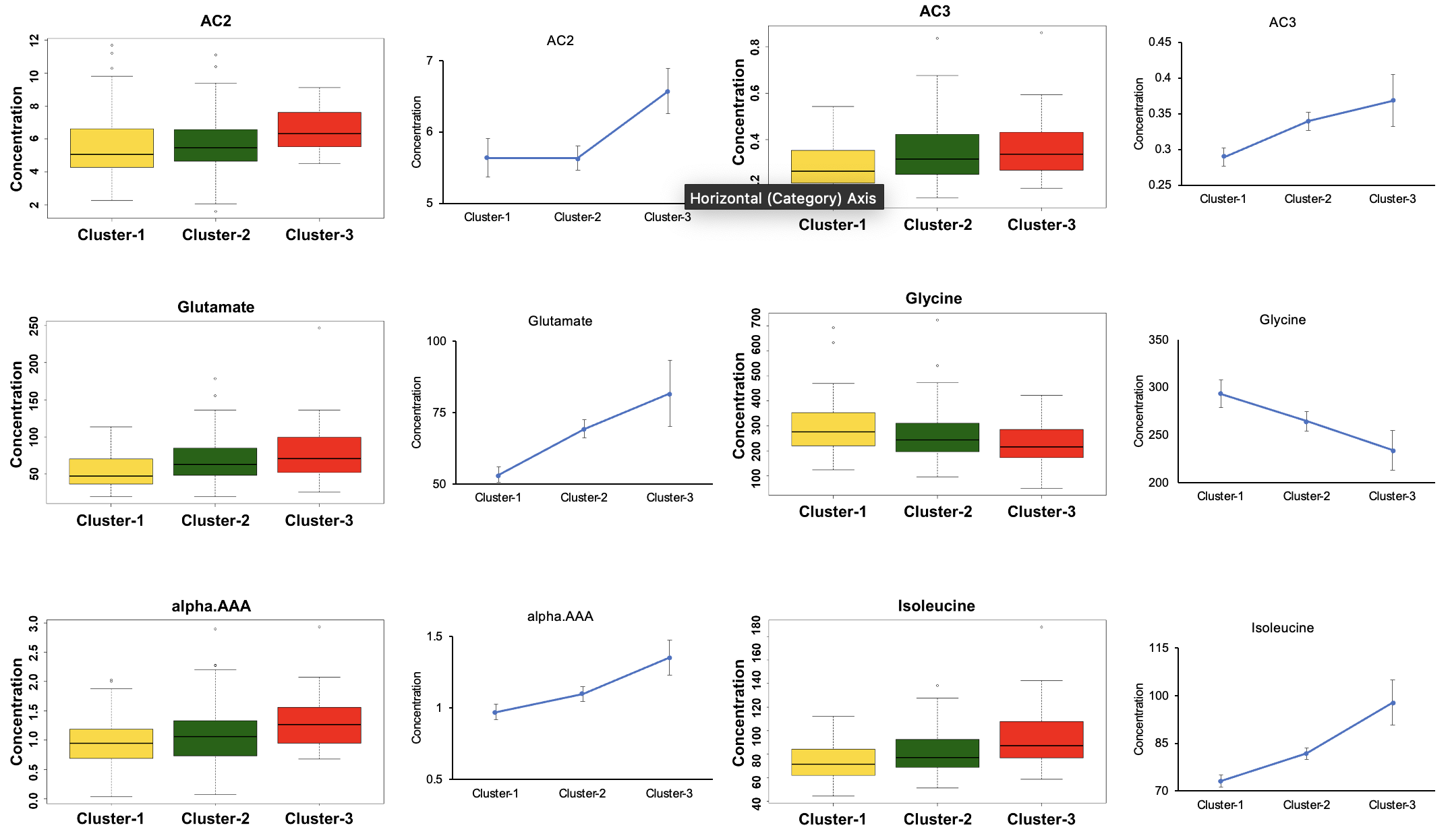


Figure S4. Concentrations of select amino acids.

**Supplemental Table 1. Infant feeding intention (IFI) scores for the cohort by lactation intensity groups at baseline.**

| **Lactation intensity groups at study baseline V1 (6-9 weeks postpartum)** | ***N*** | **Mean (SD)** | **Median (IQR)** | **P-value** |
| --- | --- | --- | --- | --- |
| Exclusive BF | 153 | 18.7 (2.4) | 20 (18, 20) | <.0001 |
| Mostly BF | 273 | 17.8 (3.0) | 19 (17, 20) |  |
| Mostly FF/Mixed/Inconsistent | 159 | 16.4 (4.0) | 17 (14, 20) |  |
| Exclusive FF | 113 | 12.4 (6.4) | 13 (8, 18) |  |

**Supplemental Table 2. SWIFT participant characteristics without diabetes at baseline by incident T2D status.**

| Characteristics | **Missing, n** | **No T2D**  **(n = 764)** | **Incident T2D**  **(n = 225)** | **P-value** |
| --- | --- | --- | --- | --- |
| **Socio-demographic** |  |  |  |  |
| Age (years) at delivery | -- | 33.1 (4.7) | 33.9 (5.1) | 0.032 |
| Race and ethnicity, n (%) | -- |  |  | 0.001 |
| Asian |  | 292 (38.2) | 70 (31.0) |  |
| Black |  | 49 (6.4) | 27 (12.0) |  |
| Hispanic |  | 218 (28.5) | 85 (37.6) |  |
| Mixed Race/Native |  | 14 (1.8) | 2 (0.9) |  |
| White |  | 191 (25.0) | 42 (18.5) |  |
| Pre-pregnancy BMI (kg/m^2^) | -- | 28.4 (6.4) | 33.7 (8.2) | <.001 |
| Family history of diabetes, n (%) |  | 352 (46.1) | 134 (59.3) | <.001 |
| **Pregnancy** |  |  |  |  |
| Parity at Index Birth, n (%) | -- |  |  | 0.001 |
| Primiparous (1 birth) |  | 285 (37.3) | 72 (31.9) |  |
| Biparous (2 births) |  | 293 (37.3) | 72 (31.9) |  |
| Multiparous (>2 births) |  | 186 (24.4) | 74 (32.7) |  |
| Gestational age at GDM diagnosis (weeks) | -- | 26.4 (6.3) | 22.3 (8.4) | <.001 |
| **Pregnancy Oral Glucose Tolerance Test** |  |  |  | <.001 |
| Fasting glucose level (mg/dl) | 2 | 90.0 (11.2) | 98.5 (12.9) | <.001 |
| 1-hour Post Glucose level (mg/dl) | -- | 196.9 (21.7) | 207.5 (25.9) | <.001 |
| 2-hour Post Glucose level (mg/dl) | 2 | 174.5 (25.4) | 181.2 (33.0) | 0.005 |
| 3-hour Post Glucose level (mg/dl) | -- | 125.2 (32.6) | 130.5 (35.3) | 0.045 |
| 3-h OGTT z-score sum of glucose values | -- | -0.4 (2.3) | 1.2 (3.1) | <.001 |
| **GDM Prenatal treatment, n (%)** | -- |  |  | <.001 |
| Diet only |  | 579 (75.8) | 100 (44.2) |  |
| Oral medications |  | 176 (23.0) | 101 (44.7) |  |
| Insulin |  | 9 (1.2) | 25 (11.1) |  |
| **Research Visit at 6-9 Weeks Postpartum (Study Baseline 2008-2011)** |  |  |  |  |
| Glucose tolerance from 2-h 75 g OGTT, n (%) | -- |  |  | <.001 |
| Normal |  | 567 (74.2) | 80 (35.4) |  |
| IFG only |  | 106 (13.9) | 74 (32.7) |  |
| IGT only |  | 65 (8.5) | 33 (14.6) |  |
| IFG + IGT |  | 26 (3.4) | 39 (17.3) |  |
| Lactation intensity categories, n (%) | -- |  |  | 0.096 |
| Exclusive lactation |  | 176 (23.0) | 35 (15.5) |  |
| Mostly lactation |  | 302 (39.5) | 94 (41.6) |  |
| Mostly formula/Mixed/Inconsistent |  | 168 (22.0) | 55 (24.3) |  |
| Exclusively formula |  | 118 (15.5) | 42 (18.6) |  |
| Physical activity, met-h/week, median (IQR) |  |  |  |  |
| Diet, intake from fat, % kcal | 6 | 24.8 (8.5) | 26.5 (7.7) | 0.006 |
| Diet, intake from fiber, kcal | 6 | 1.06 (0.45) | 1.02 (0.36) | 0.110 |
| **Anthropometry** |  |  |  |  |
| Weight (kg) | -- | 74.0 (16.8) | 86.3 (22.6) | <.001 |
| Height (cm) | -- | 159.3 (6.7) | 159.9 (7.3) | 0.323 |
| BMI (kg/m^2^) | -- | 29.1 (6.0) | 33.5 (7.5) | <.001 |
| BMI Categories, n (%): | -- |  |  | <.001 |
| Normal (< 25 kg/m^2^) |  | 202 (26.4) | 25 (11.1) |  |
| Overweight (25 to 29.9 kg/m^2^) |  | 289 (37.8) | 52 (12.4) |  |
| Obese (≥30 kg/m^2^) |  | 273 (35.7) | 149 (65.9) |  |
| Waist Circumference (cm) | 7 | 87.7 (12.7) | 97.4 (14.5) | <.001 |
| **Plasma, 2-h 75 gram Research OGTT**  **at 6-9 weeks postpartum** |  |  |  |  |
| Fasting glucose (mg/dL) | -- | 92.9 (7.7) | 100.6 (10.3) | <.001 |
| 2-h Post-load glucose (mg/dL) | -- | 107.8 (25.9) | 127.8 (29.5) | <.001 |
| Fasting insulin (µU/ml), median (IQR) | 4 | 17.7 (12.4, 25.0) | 26.5 (17.8, 38.8) | <.001 |
| 2-h Post-load insulin (µU/mL), median (IQR) | 1 | 75.6 (49.7, 109.5) | 108.6 (71.2, 153.4) | <.001 |
| HOMA-IR, Median (IQR) | 4 | 4.0 (2.7, 5.9) | 6.6 (4.4, 10.1) | <.001 |
| HOMA-B, Median (IQR) | 4 | 221 (160, 306) | 268 (179, 374) | <.001 |
| Fasting Triglycerides (mg/dL), Median  (IQR) | 7 | 94 (67, 143) | 114 (79, 176) | <.001 |
| Fasting HDL-C (mg/dL) | 7 | 53.9 (14.2) | 48.6 (12.8) | <.001 |
| Fasting LDL-C (mg/dL) | 7 | 127.1 (31.0) | 122.1 (30.0) | 0.029 |
| Fasting Adiponectin (ug/mL), median (IQR) | 7 | 7.2 (5.7, 9.2) | 6.4 (5.0, 8.0) | <.001 |
| Fasting Leptin (ng/mL) | 7 | 23.8 (14.1, 35.8) | 33.0 (22.7, 43.0) | <.001 |
| **During Post-baseline to End of Follow Up through 2020** |  |  |  |  |
| Follow up Time (months), Median (IQR) | -- | 94.1 (53.7, 111.3) | 35.2 (13.3, 81.5) | <.001 |

IFG = impaired fasting glucose; IGT = impaired glucose tolerance; 2-hour post-load glucose.

(P-values: Chi-squared used for categorical variables, ANOVA used for continuous, and Kruskal-Wallis used for medians). Mean (SD) or n (%) unless otherwise noted as Median (IQR) for skewedness of variables.

**Supplemental Table 3. Distribution of women across clusters by glycemic control and GDM treatment.**

| **Groups** | **Cluster-β**  **(n = 81)** | **Cluster-mixed**  **(n = 120)** | **Cluster-IR**  **(n = 24)** | **P-value** |
| --- | --- | --- | --- | --- |
| Diet only x optimal glycemic control | 27 (37.0) | 33 (31.4) | 2 (13.3) | 0.053 |
| Diet only x suboptimal glycemic control | 7 (9.6) | 17 (16.2) | 6 (40.0) |  |
| Medication x optimal glycemic control | 20 (27.4) | 21 (20.0) | 5 (33.3) |  |
| Medication x suboptimal glycemic control | 19 (26.0) | 34 (32.4) | 2 (13.3) |  |

Data presented as frequency (%).
